# 2-Methoxy­benzaldehyde 2,4-dinitro­phenyl­hydrazone

**DOI:** 10.1107/S1600536809000038

**Published:** 2009-01-08

**Authors:** Hoong-Kun Fun, Reza Kia, Hadi Kargar

**Affiliations:** a X-ray Crystallography Unit, School of Physics, Universiti Sains Malaysia, 11800 USM, Penang, Malaysia; b Department of Chemistry, School of Science, Payame Noor University (PNU), Ardakan, Yazd, Iran

## Abstract

In the title compound, C_14_H_12_N_4_O_5_, an intra­molecular N—H⋯O hydrogen bond generates an *S*(6) ring motif. The dihedral angle between the two benzene rings is 3.91 (3)°, which shows the mol­ecule is almost planar. The *para*-nitro group is twisted from the benzene ring to which it is attached, making a dihedral angle of 8.50 (9)°. In the crystal structure, mol­ecules are linked together by inter­molecular C—H⋯O and inter­molecular three-centred O⋯O [2.8646 (12)–2.9213 (11) Å] and O⋯N [3.0518 (11) Å] inter­actions. The crystal structure is further stabilized by inter­molecular π–π inter­actions [centroid-to-centroid distances 3.5708 (6)–3.9728 (12) Å].

## Related literature

For general background, see: Lamberton *et al.* (1974 ▶); Zegota (1999 ▶); Cordis *et al.* (1998 ▶); Zlotorzynska & Lai (1999 ▶); Niknam *et al.* (2005 ▶); Guillaumont & Nakamura (2000 ▶); Raj & Kurup (2006 ▶). For biological applications, see: Okabe *et al.* (1993 ▶). Standard bond-length data are given in: Allen *et al.* (1987 ▶). For details of the classification of ring motifs, see: Bernstein *et al.* (1995 ▶).
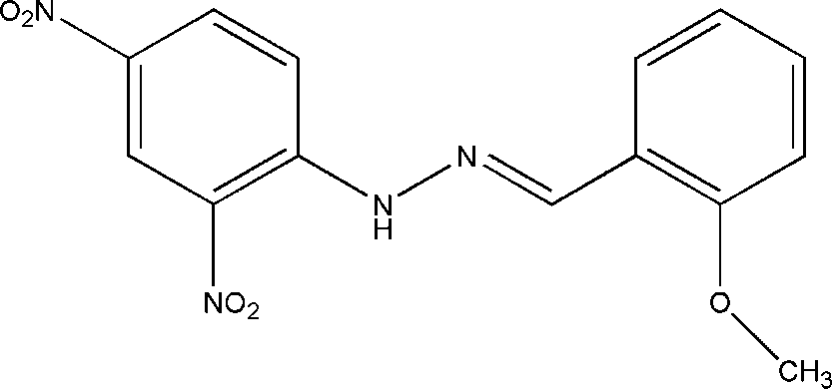


## Experimental

### 

#### Crystal data

C_14_H_12_N_4_O_5_
*M* *_r_* = 316.28
Triclinic, 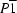

*a* = 7.0315 (1) Å
*b* = 7.6205 (2) Å
*c* = 14.1896 (4) Å
α = 98.048 (1)°
β = 97.064 (1)°
γ = 109.467 (1)°
*V* = 697.99 (3) Å^3^
*Z* = 2
Mo *K*α radiation
μ = 0.12 mm^−1^
*T* = 100.0 (1) K
0.57 × 0.23 × 0.10 mm

#### Data collection

Bruker SMART APEXII CCD area-detector diffractometer
Absorption correction: multi-scan (*SADABS*; Bruker, 2005 ▶) *T*_min_ = 0.936, *T*_max_ = 0.988
14423 measured reflections
5023 independent reflections
4442 reflections with *I* > 2σ(*I*)
*R*_int_ = 0.021

#### Refinement

*R*[*F* ^2^ > 2σ(*F* ^2^)] = 0.045
*wR*(*F* ^2^) = 0.126
*S* = 1.06
5023 reflections
213 parameters
H atoms treated by a mixture of independent and constrained refinement
Δρ_max_ = 0.52 e Å^−3^
Δρ_min_ = −0.25 e Å^−3^

### 

Data collection: *APEX2* (Bruker, 2005 ▶); cell refinement: *APEX2*; data reduction: *SAINT* (Bruker, 2005 ▶); program(s) used to solve structure: *SHELXTL* (Sheldrick, 2008 ▶); program(s) used to refine structure: *SHELXTL*; molecular graphics: *SHELXTL*; software used to prepare material for publication: *SHELXTL* and *PLATON* (Spek, 2003 ▶).

## Supplementary Material

Crystal structure: contains datablocks global, I. DOI: 10.1107/S1600536809000038/pk2139sup1.cif

Structure factors: contains datablocks I. DOI: 10.1107/S1600536809000038/pk2139Isup2.hkl

Additional supplementary materials:  crystallographic information; 3D view; checkCIF report

## Figures and Tables

**Table 1:** Hydrogen-bond geometry (Å, °)

*D*—H⋯*A*	*D*—H	H⋯*A*	*D*⋯*A*	*D*—H⋯*A*
N2—H1N2⋯O2	0.864 (15)	2.029 (15)	2.6253 (11)	125.4 (13)
N2—H1N2⋯O2^i^	0.864 (15)	2.599 (15)	3.3475 (11)	145.6 (13)
C2—H2*A*⋯O4^ii^	0.93	2.44	3.3113 (12)	155
C5—H5*A*⋯O2^iii^	0.93	2.60	3.3184 (11)	135

Symmetry codes: (i) 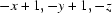; (ii) 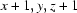; (iii) 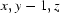.

**Fig. 1.**
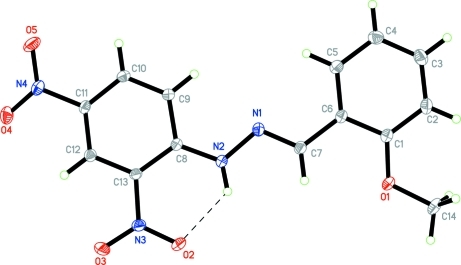
The molecular structure of the title compound, showing 50% probability displacement ellipsoids and the atomic numbering scheme. Hydrogen bond is shown as a dashed line.

**Fig. 2.**
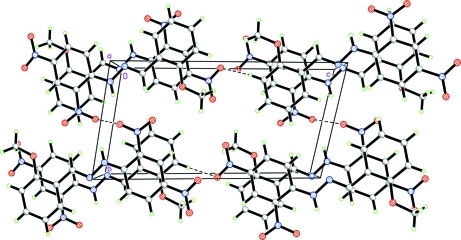
The crystal packing of the title compound, viewed down the a axis, showing the molecules are linked through intermolecular C—H···O and O···O interactions and also are stacked along the a axis. Intermolecular interactions are shown as dashed lines.

## supplementary crystallographic information

### Comment

2,4-Dinitrophenylhydrazones play a more important role as stabilizers for the detection, characterization and protection of carbonyl groups than phenylhydrazones (Niknam *et al.*, 2005). 2,4-Dinitrophenylhydrazone derivatives are widely used in various forms of analytical chemistry (Lamberton *et al.*, 1974; Zegota, 1999; Cordis *et al.*, 1998; Zlotorzynska & Lai, 1999) and are also used as dyes (Guillaumont & Nakamura, 2000). They are also found to have versatile coordinating abilities towards different metal ions (Raj & Kurup, 2006). In addition, some phenylhydrazone derivatives have been shown to be potentially DNA-damaging and mutagenic agents (Okabe *et al.,* 1993). For these reasons, the structure of the title compound is reported here.

Bond lengths in the title compound (Fig. 1) are normal (Allen *et al.*, 1987). An intramolecular N—H···O hydrogen bond generates an *S(6)* ring motif (Bernstein *et al.*, 1995). The molecule is nearly planar, with a maximum deviation from the mean plane of -0.3464 (8) Å for atom O4 which is due to the intermolecular three-centered O···O and O···N interactions. The dihedral angle between the two benzene rings is 4.63 (1)°. Interesting features of the crystal structure include intermolecular three-centered O2···O2^i^ [2.8646 (12) Å; (i) 1 - *x*, 1 - *y*, -*z*], O4···O4^iv^ [2.8646 (12) Å; (iv) code: -*x*, -*y*, -1 - *z*], and O4···N4^ii^ [3.0518 (11) Å] interactions. The molecules are also linked by C—H···O hydrogen bonds (Table 1), and by intermolecular π–π interactions giving centroid–centroid distances for rings C1–C6 (*Cg*1) and C8–C13 (*Cg*2) of 3.5708 (6) Å and 3.9728 (12) Å [*Cg*1···*Cg*2^v^; (v) -*x*, -*y*, -*z* and *Cg*1···*Cg*2^vi^; (vi) 1 - *x*, -*y*, -*z*] (interplanar spacings are 3.3080 (4) and 3.3691 (4) Å respectively (Fig. 2).

### Experimental

The title compound was synthesized based on the reported procedure (Okabe *et al.* 1993) except that 3-methoxybenzaldehyde (1 mmol, 136 mg) was used instead. Single crystals suitable for X-ray diffraction analysis were grown by slow evaporation of a saturated solution of the resulted compound in ethanol.

### Refinement

N-bound H atom was located from the difference Fourier map and refined freely. The remaining H atoms were placed in calculated positions (C—H = 0.93–0.96 Å) and refined using a riding model, with U_iso_(H) = 1.2 or 1.5U_eq_(C). A rotating group model was applied to the methyl group.

### Crystal data

**Table:**

C_14_H_12_N_4_O_5_	*Z* = 2
*M**_r_* = 316.28	*F*(000) = 328
Triclinic, *P*1	*D*_x_ = 1.505 Mg m^−^^3^
Hall symbol: -P 1	Mo *K*α radiation, λ = 0.71073 Å
*a* = 7.0315 (1) Å	Cell parameters from 6651 reflections
*b* = 7.6205 (2) Å	θ = 2.9–40.3°
*c* = 14.1896 (4) Å	µ = 0.12 mm^−^^1^
α = 98.048 (1)°	*T* = 100 K
β = 97.064 (1)°	Block, orange
γ = 109.467 (1)°	0.57 × 0.23 × 0.10 mm
*V* = 697.99 (3) Å^3^	

### Data collection

**Table:**

Bruker SMART APEXII CCD area-detector diffractometer	5023 independent reflections
Radiation source: fine-focus sealed tube	4442 reflections with *I* > 2σ(*I*)
graphite	*R*_int_ = 0.021
φ and ω scans	θ_max_ = 32.5°, θ_min_ = 1.5°
Absorption correction: multi-scan (*SADABS*; Bruker, 2005)	*h* = −10→10
*T*_min_ = 0.936, *T*_max_ = 0.988	*k* = −11→11
14423 measured reflections	*l* = −21→21

### Refinement

**Table:**

Refinement on *F*^2^	Primary atom site location: structure-invariant direct methods
Least-squares matrix: full	Secondary atom site location: difference Fourier map
*R*[*F*^2^ > 2σ(*F*^2^)] = 0.045	Hydrogen site location: inferred from neighbouring sites
*wR*(*F*^2^) = 0.126	H atoms treated by a mixture of independent and constrained refinement
*S* = 1.06	*w* = 1/[σ^2^(*F*_o_^2^) + (0.0683*P*)^2^ + 0.1626*P*] where *P* = (*F*_o_^2^ + 2*F*_c_^2^)/3
5023 reflections	(Δ/σ)_max_ < 0.001
213 parameters	Δρ_max_ = 0.52 e Å^−^^3^
0 restraints	Δρ_min_ = −0.24 e Å^−^^3^

### Special details

**Table:**

Experimental. The low-temperature data was collected with the Oxford Cyrosystem Cobra low-temperature attachment.
Geometry. All esds (except the esd in the dihedral angle between two l.s. planes) are estimated using the full covariance matrix. The cell esds are taken into account individually in the estimation of esds in distances, angles and torsion angles; correlations between esds in cell parameters are only used when they are defined by crystal symmetry. An approximate (isotropic) treatment of cell esds is used for estimating esds involving l.s. planes.
Refinement. Refinement of F^2^ against ALL reflections. The weighted R-factor wR and goodness of fit S are based on F^2^, conventional R-factors R are based on F, with F set to zero for negative F^2^. The threshold expression of F^2^ > 2sigma(F^2^) is used only for calculating R-factors(gt) etc. and is not relevant to the choice of reflections for refinement. R-factors based on F^2^ are statistically about twice as large as those based on F, and R- factors based on ALL data will be even larger.

### Fractional atomic coordinates and isotropic or equivalent isotropic displacement parameters (Å^2^)

**Table:**

	*x*	*y*	*z*	*U*_iso_*/*U*_eq_	
O1	0.52355 (11)	0.19077 (9)	0.30740 (5)	0.01789 (14)	
O2	0.31793 (12)	0.47602 (10)	−0.06604 (5)	0.02132 (15)	
O3	0.20422 (12)	0.51054 (10)	−0.20851 (5)	0.02350 (16)	
O4	−0.18137 (11)	−0.00408 (11)	−0.45869 (5)	0.02104 (15)	
O5	−0.19171 (12)	−0.28928 (10)	−0.44929 (5)	0.02410 (16)	
N1	0.27092 (11)	0.00256 (11)	0.02604 (5)	0.01410 (14)	
N2	0.26656 (12)	0.13732 (11)	−0.02830 (5)	0.01416 (14)	
N3	0.22761 (12)	0.40930 (10)	−0.15138 (6)	0.01487 (15)	
N4	−0.14093 (12)	−0.11921 (11)	−0.41450 (5)	0.01611 (15)	
C1	0.46732 (13)	0.00043 (12)	0.27560 (6)	0.01377 (15)	
C2	0.48758 (14)	−0.12628 (13)	0.33510 (6)	0.01710 (17)	
H2A	0.5476	−0.0815	0.4001	0.021*	
C3	0.41767 (15)	−0.31935 (14)	0.29676 (7)	0.01947 (18)	
H3A	0.4306	−0.4035	0.3365	0.023*	
C4	0.32856 (14)	−0.38821 (13)	0.19954 (7)	0.01804 (17)	
H4A	0.2814	−0.5177	0.1745	0.022*	
C5	0.31052 (13)	−0.26234 (12)	0.14015 (6)	0.01530 (16)	
H5A	0.2514	−0.3085	0.0751	0.018*	
C6	0.38009 (12)	−0.06701 (12)	0.17671 (6)	0.01284 (15)	
C7	0.36703 (13)	0.06671 (12)	0.11394 (6)	0.01371 (15)	
H7A	0.4276	0.1967	0.1373	0.016*	
C8	0.17242 (12)	0.07996 (12)	−0.12228 (6)	0.01178 (15)	
C9	0.09189 (13)	−0.11672 (12)	−0.16426 (6)	0.01378 (15)	
H9A	0.1056	−0.2038	−0.1266	0.017*	
C10	−0.00529 (13)	−0.18102 (12)	−0.25882 (6)	0.01405 (15)	
H10A	−0.0547	−0.3101	−0.2852	0.017*	
C11	−0.02975 (12)	−0.05112 (12)	−0.31553 (6)	0.01291 (15)	
C12	0.04681 (12)	0.14065 (12)	−0.27956 (6)	0.01311 (15)	
H12A	0.0307	0.2253	−0.3184	0.016*	
C13	0.14893 (12)	0.20609 (11)	−0.18400 (6)	0.01222 (15)	
C14	0.64623 (15)	0.27271 (14)	0.40200 (7)	0.01993 (18)	
H14A	0.6876	0.4084	0.4120	0.030*	
H14B	0.7657	0.2377	0.4074	0.030*	
H14C	0.5675	0.2269	0.4500	0.030*	
H1N2	0.329 (2)	0.256 (2)	−0.0027 (11)	0.035 (4)*	

### Atomic displacement parameters (Å^2^)

**Table:**

	*U*^11^	*U*^22^	*U*^33^	*U*^12^	*U*^13^	*U*^23^
O1	0.0227 (3)	0.0151 (3)	0.0117 (3)	0.0049 (2)	−0.0022 (2)	−0.0014 (2)
O2	0.0294 (4)	0.0137 (3)	0.0151 (3)	0.0053 (3)	−0.0036 (3)	−0.0028 (2)
O3	0.0317 (4)	0.0130 (3)	0.0239 (4)	0.0077 (3)	−0.0032 (3)	0.0055 (3)
O4	0.0218 (3)	0.0268 (4)	0.0147 (3)	0.0103 (3)	−0.0016 (2)	0.0048 (3)
O5	0.0274 (4)	0.0189 (3)	0.0185 (3)	0.0055 (3)	−0.0040 (3)	−0.0063 (3)
N1	0.0153 (3)	0.0149 (3)	0.0115 (3)	0.0049 (2)	0.0015 (2)	0.0030 (2)
N2	0.0173 (3)	0.0123 (3)	0.0109 (3)	0.0042 (3)	−0.0004 (2)	0.0014 (2)
N3	0.0162 (3)	0.0115 (3)	0.0158 (3)	0.0051 (2)	0.0007 (3)	0.0007 (2)
N4	0.0144 (3)	0.0190 (3)	0.0126 (3)	0.0052 (3)	−0.0001 (2)	−0.0002 (3)
C1	0.0130 (3)	0.0156 (4)	0.0116 (3)	0.0044 (3)	0.0017 (3)	0.0018 (3)
C2	0.0164 (4)	0.0212 (4)	0.0130 (4)	0.0057 (3)	0.0011 (3)	0.0049 (3)
C3	0.0199 (4)	0.0207 (4)	0.0195 (4)	0.0072 (3)	0.0041 (3)	0.0091 (3)
C4	0.0191 (4)	0.0142 (4)	0.0199 (4)	0.0042 (3)	0.0047 (3)	0.0040 (3)
C5	0.0151 (3)	0.0150 (4)	0.0136 (4)	0.0032 (3)	0.0024 (3)	0.0014 (3)
C6	0.0123 (3)	0.0145 (3)	0.0108 (3)	0.0040 (3)	0.0017 (3)	0.0018 (3)
C7	0.0145 (3)	0.0138 (3)	0.0118 (3)	0.0042 (3)	0.0019 (3)	0.0016 (3)
C8	0.0120 (3)	0.0124 (3)	0.0103 (3)	0.0042 (3)	0.0013 (3)	0.0010 (3)
C9	0.0160 (3)	0.0113 (3)	0.0128 (4)	0.0039 (3)	0.0015 (3)	0.0020 (3)
C10	0.0151 (3)	0.0111 (3)	0.0136 (4)	0.0030 (3)	0.0014 (3)	0.0001 (3)
C11	0.0119 (3)	0.0144 (3)	0.0103 (3)	0.0034 (3)	0.0000 (3)	0.0007 (3)
C12	0.0128 (3)	0.0139 (3)	0.0124 (3)	0.0051 (3)	0.0008 (3)	0.0022 (3)
C13	0.0128 (3)	0.0100 (3)	0.0129 (3)	0.0039 (3)	0.0007 (3)	0.0007 (3)
C14	0.0188 (4)	0.0225 (4)	0.0131 (4)	0.0048 (3)	−0.0019 (3)	−0.0032 (3)

### Geometric parameters (Å, °)

**Table:**

O1—C1	1.3618 (11)	C4—C5	1.3883 (13)
O1—C14	1.4344 (11)	C4—H4A	0.9300
O2—N3	1.2456 (10)	C5—C6	1.4001 (12)
O3—N3	1.2274 (10)	C5—H5A	0.9300
O4—N4	1.2329 (10)	C6—C7	1.4617 (12)
O5—N4	1.2328 (10)	C7—H7A	0.9300
N1—C7	1.2858 (11)	C8—C9	1.4224 (11)
N1—N2	1.3736 (10)	C8—C13	1.4227 (11)
N2—C8	1.3545 (10)	C9—C10	1.3692 (11)
N2—H1N2	0.864 (16)	C9—H9A	0.9300
N3—C13	1.4435 (11)	C10—C11	1.3998 (12)
N4—C11	1.4520 (11)	C10—H10A	0.9300
C1—C2	1.3985 (12)	C11—C12	1.3730 (12)
C1—C6	1.4092 (11)	C12—C13	1.3912 (11)
C2—C3	1.3894 (14)	C12—H12A	0.9300
C2—H2A	0.9300	C14—H14A	0.9600
C3—C4	1.3916 (13)	C14—H14B	0.9600
C3—H3A	0.9300	C14—H14C	0.9600
			
C1—O1—C14	118.36 (7)	C1—C6—C7	119.95 (7)
C7—N1—N2	115.65 (7)	N1—C7—C6	119.25 (8)
C8—N2—N1	118.87 (7)	N1—C7—H7A	120.4
C8—N2—H1N2	121.6 (10)	C6—C7—H7A	120.4
N1—N2—H1N2	119.4 (10)	N2—C8—C9	119.67 (7)
O3—N3—O2	122.20 (7)	N2—C8—C13	123.82 (7)
O3—N3—C13	119.09 (7)	C9—C8—C13	116.51 (7)
O2—N3—C13	118.71 (7)	C10—C9—C8	121.61 (8)
O5—N4—O4	123.66 (8)	C10—C9—H9A	119.2
O5—N4—C11	118.19 (7)	C8—C9—H9A	119.2
O4—N4—C11	118.15 (7)	C9—C10—C11	119.52 (8)
O1—C1—C2	123.98 (8)	C9—C10—H10A	120.2
O1—C1—C6	115.86 (7)	C11—C10—H10A	120.2
C2—C1—C6	120.14 (8)	C12—C11—C10	121.62 (8)
C3—C2—C1	119.74 (8)	C12—C11—N4	118.64 (7)
C3—C2—H2A	120.1	C10—C11—N4	119.74 (7)
C1—C2—H2A	120.1	C11—C12—C13	118.80 (8)
C2—C3—C4	120.73 (8)	C11—C12—H12A	120.6
C2—C3—H3A	119.6	C13—C12—H12A	120.6
C4—C3—H3A	119.6	C12—C13—C8	121.90 (7)
C5—C4—C3	119.58 (8)	C12—C13—N3	115.84 (7)
C5—C4—H4A	120.2	C8—C13—N3	122.26 (7)
C3—C4—H4A	120.2	O1—C14—H14A	109.5
C4—C5—C6	120.96 (8)	O1—C14—H14B	109.5
C4—C5—H5A	119.5	H14A—C14—H14B	109.5
C6—C5—H5A	119.5	O1—C14—H14C	109.5
C5—C6—C1	118.84 (8)	H14A—C14—H14C	109.5
C5—C6—C7	121.21 (7)	H14B—C14—H14C	109.5
			
C7—N1—N2—C8	178.35 (7)	C13—C8—C9—C10	−0.77 (12)
C14—O1—C1—C2	12.61 (13)	C8—C9—C10—C11	−1.15 (13)
C14—O1—C1—C6	−168.81 (8)	C9—C10—C11—C12	1.96 (13)
O1—C1—C2—C3	177.25 (8)	C9—C10—C11—N4	−177.18 (8)
C6—C1—C2—C3	−1.27 (13)	O5—N4—C11—C12	173.05 (8)
C1—C2—C3—C4	0.36 (14)	O4—N4—C11—C12	−7.77 (12)
C2—C3—C4—C5	0.38 (14)	O5—N4—C11—C10	−7.78 (12)
C3—C4—C5—C6	−0.21 (14)	O4—N4—C11—C10	171.39 (8)
C4—C5—C6—C1	−0.69 (13)	C10—C11—C12—C13	−0.75 (13)
C4—C5—C6—C7	178.17 (8)	N4—C11—C12—C13	178.40 (7)
O1—C1—C6—C5	−177.21 (7)	C11—C12—C13—C8	−1.28 (13)
C2—C1—C6—C5	1.43 (13)	C11—C12—C13—N3	179.24 (7)
O1—C1—C6—C7	3.91 (12)	N2—C8—C13—C12	−178.46 (8)
C2—C1—C6—C7	−177.45 (8)	C9—C8—C13—C12	2.01 (12)
N2—N1—C7—C6	−179.67 (7)	N2—C8—C13—N3	0.98 (13)
C5—C6—C7—N1	7.29 (13)	C9—C8—C13—N3	−178.55 (7)
C1—C6—C7—N1	−173.86 (8)	O3—N3—C13—C12	−1.30 (12)
N1—N2—C8—C9	−3.93 (12)	O2—N3—C13—C12	178.99 (8)
N1—N2—C8—C13	176.55 (8)	O3—N3—C13—C8	179.23 (8)
N2—C8—C9—C10	179.68 (8)	O2—N3—C13—C8	−0.48 (12)

### Hydrogen-bond geometry (Å, °)

**Table:**

*D*—H···*A*	*D*—H	H···*A*	*D*···*A*	*D*—H···*A*
N2—H1N2···O2	0.864 (15)	2.029 (15)	2.6253 (11)	125.4 (13)
N2—H1N2···O2^i^	0.864 (15)	2.599 (15)	3.3475 (11)	145.6 (13)
C2—H2A···O4^ii^	0.93	2.44	3.3113 (12)	155
C5—H5A···O2^iii^	0.93	2.60	3.3184 (11)	135

Symmetry codes: (i) −*x*+1, −*y*+1, −*z*; (ii) *x*+1, *y*, *z*+1; (iii) *x*, *y*−1, *z*.
